# Multiple Temperature-Sensing Behavior of Green and Red Upconversion Emissions from Stark Sublevels of Er^3+^

**DOI:** 10.3390/s151229839

**Published:** 2015-12-10

**Authors:** Baosheng Cao, Jinlei Wu, Xuehan Wang, Yangyang He, Zhiqing Feng, Bin Dong

**Affiliations:** School of Physics and Materials Engineering, Dalian Nationalities University, Dalian 116600, China; bscao@dlnu.edu.cn (B.C.); mrwu888@live.cn (J.W.); xhanwang@tom.com (X.W.); yyhe@dlnu.edu.cn (Y.H.); fzq@dlnu.edu.cn (Z.F.)

**Keywords:** temperature sensing, upconversion emissions, Stark sublevel, rare earth, sensitivity

## Abstract

Upconversion luminescence properties from the emissions of Stark sublevels of Er^3+^ were investigated in Er^3+^-Yb^3+^-Mo^6+^-codoped TiO_2_ phosphors in this study. According to the energy levels split from Er^3+^, green and red emissions from the transitions of four coupled energy levels, ^2^H_11/2(I)_/^2^H_11/2(II)_, ^4^S_3/2(I)_/^4^S_3/2(II)_, ^4^F_9/2(I)_/^4^F_9/2(II)_, and ^2^H_11/2(I)_ + ^2^H_11/2(II)_/^4^S_3/2(I)_ + ^4^S_3/2(II)_, were observed under 976 nm laser diode excitation. By utilizing the fluorescence intensity ratio (FIR) technique, temperature-dependent upconversion emissions from these four coupled energy levels were analyzed at length. The optical temperature-sensing behaviors of sensing sensitivity, measurement error, and operating temperature for the four coupled energy levels are discussed, all of which are closely related to the energy gap of the coupled energy levels, FIR value, and luminescence intensity. Experimental results suggest that Er^3+^-Yb^3+^-Mo^6+^-codoped TiO_2_ phosphor with four pairs of energy levels coupled by Stark sublevels provides a new and effective route to realize multiple optical temperature-sensing through a wide range of temperatures in an independent system.

## 1. Introduction

Optical temperature-sensing devices have been widely researched to promote their application in electrical power stations, oil refineries, coal mines, and fire detection, as they have been shown to overcome the interference of strong electromagnetic noise, hazardous sparks, or corrosive environments inaccessible to traditional temperature-measurement methods such as thermocouple detectors [1,2,3,4,5]. Sensors built based on the fluorescence intensity ratio (FIR) technique have attracted particular attention due to their ability to reduce dependence on measurement conditions and improve accuracy and resolution. FIR functions independent of fluorescence loss or fluctuations in excitation intensity can be applied to fluorescence systems in which two closely spaced energy levels with separations of the order of thermal energy are involved, following a Boltzmann-type population distribution [1,6,7]. Optical temperature sensors using the FIR technique are mainly focused on fluoride and oxides matrixes [8,9,10,11,12,13,14]. The fluoride matrixes possesses higher fluorescence efficiency and lower excitation power; however, the maximum operating temperature is usually low. On the contrary, the oxides matrices can operate at high temperature, although the fluorescence intensity is lower.

Upconversion emissions of rare earth ion-doped materials are typically utilized to realize FIR measurement because of the large amount of coupled energy levels in many rare earth ions and the easily accessible upconversion luminescence with near-infrared radiation from low-cost, commercially available diodes. Xu *et al.* [8], for example, reported the FIR of Ho^3+^ using two blue emissions from coupled energy levels of ^5^G_6_/^5^F_1_ and ^5^F_2,3_/^3^K_8_ and found that Ho^3+^-Yb^3+^-codoped CaWO_4_ possessed higher absolute sensitivity due to a larger energy gap between the thermally coupled ^5^G_6_/^5^F_1_ and ^5^F_2,3_/^3^K_8_ levels of Ho^3+^ ions. The paired energy levels of ^3^F_2_ and ^3^F_3_ in Tm^3+^ ions have also been used to investigate temperature-dependent red upconversion emissions and corresponding FIR properties [9]. The FIR properties of green upconversion emissions ascribed to paired energy levels of ^2^H_11/2_ and ^4^S_3/2_ in Er^3+^-doped materials, in particular, have been quite widely studied [10,11,12,13,14].

In addition to the intrinsic thermally coupled energy levels of rare earth ions, the pair energy levels of Stark sublevels can also be thermally coupled and used to investigate FIR *versus* temperature characteristics [15,16,17,18]. Baxter *et al.* [17], for example, used the coupled energy levels of ^2^F_5/2(a)_ and ^2^F_5/2(b)_ by Stark split of ^2^F_5/2_ levels in Yb^3+^ ions to study FIR properties of Yb^3+^-doped silica fiber. Feng *et al.* [18] investigated the FIR properties of Er^3+^-doped fluoride glass using coupled Stark sublevels of ^4^S_3/2(1)_ and ^4^S_3/2(2)_ in Er^3+^ ions.

In this study, four thermally coupled energy levels of Er^3+^ ions based on the Stark sublevels were simultaneously observed in Er^3+^-Yb^3+^-Mo^6+^-codoped TiO_2_ phosphors. FIR properties of the four coupled energy levels from green and red emissions in Er^3+^-Yb^3+^-Mo^6+^-codoped TiO_2_ phosphors were studied as a function of temperature in the range of 307–673 K. The effects of the energy gap of thermally coupled energy levels, FIR value, and upconversion emission intensity on the sensitivity and accuracy of the optical temperature sensor are discussed in an effort to explore potential developments in optical temperature-sensor technology based on different FIR routes in an independent system.

## 2. Experimental Section

The sol-gel method was used to prepare Er^3+^-Yb^3+^-Mo^6+^-codoped TiO_2_ phosphors. The rare earth nitrates Er(NO_3_)_3_·5H_2_O (99.99%) and Yb(NO_3_)_3_·5H_2_O (99.99%) were purchased from Aladdin. Other chemicals including Iso-Propanol (i-PrOH), *n*-butyl titanate (Ti(OBu)_4_), acetylacetone (AcAc), and concentrated nitric acid (HNO_3_) were purchased from Sinopharm Chemical Reagent Co., Ltd. (Shanghai, China). All chemicals are of analytical reagent and were used without any further purification. i-PrOH was first added as a solvent to modified titanium(IV) *n*-butoxide by facilitating a chelating reaction between Ti(OBu)_4_ and AcAc under agitation for 1 h at room temperature. Next, a mixture of deionized water, i-PrOH, and HNO_3_ was slowly added into the solution. The mixed solution was stirred for 6 h to form a clear and stable sol. The molar ratios of Ti(OBu)_4_, AcAc, H_2_O, and HNO_3_ were 3:3:6:1. Finally, Er, Mo, and Yb ions were introduced by adding Er(NO_3_)_3_·5H_2_O, (NH_4_)_6_Mo_7_O_24_·5H_2_O, and Yb(NO_3_)_3_·5H_2_O in the molar ratio of 2:2:20:100 for Er:Mo:Yb:Ti. The codoped sols were dried at 373 K for 8 h to remove the solvent. The xerogels were then heated at a rate of 4 K/min and maintained at the sintering temperature of 1073 K for 1 h, then cooled to room temperature in the furnace. The sintered 2 mol % Er^3+^–20 mol % Yb^3+^–2 mol % Mo^6+^-codoped TiO_2_ phosphors were finally milled into powders for structural analysis and spectral measurement.

The phase structures of Er^3+^-Yb^3+^-Mo^6+^-codoped TiO_2_ phosphor samples were analyzed by SHIMADZU XRD-6000 X-ray diffractormeter (XRD) with Cu-Kα radiation. A homemade temperature control system, which was composed of a small stove and an intelligent digital-display-type temperature control instrument, was used to adjust sample temperature from 307 to 673 K, at measurement and control accuracy of about ±0.5 K. Temperature-dependent upconversion emissions from each sample were focused onto a Jobin Yvon iHr550 monochromator and detected with a CR131 photomultiplier tube by 976 nm laser diode (LD) excitation. The LD pump current varied from 0 to 2 A, and the spectral resolution of the experimental set-up was 0.1 nm.

## 3. Results and Discussion

**Figure 1:**
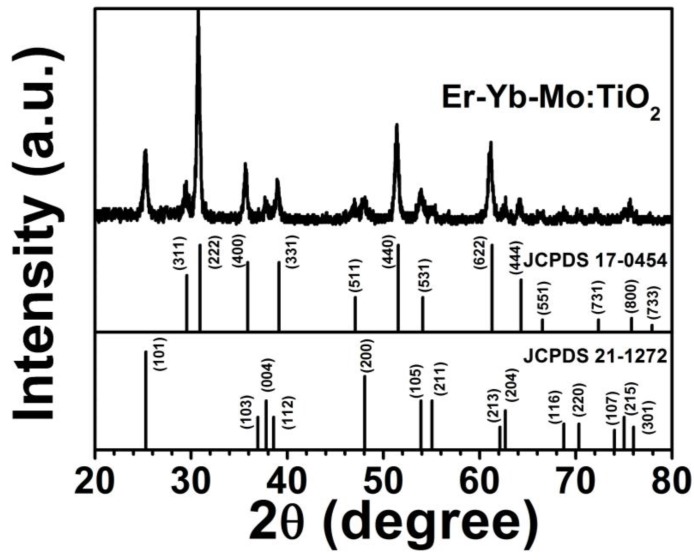
XRD pattern of Er^3+^-Yb^3+^-Mo^6+^ codoped TiO_2_.

Figure 1 shows XRD patterns of the Er^3+^-Yb^3+^-Mo^6+^-codoped TiO_2_ phosphor samples. The XRD pattern observed was characteristic of the anatase phase of TiO_2_ (JCPDS No. 21-1272) and the face-centered cubic phase of Yb_2_Ti_2_O_7_ (JCPDS No. 17-0454) referenced below. There was no diffraction peak of Mo compounds, and the main diffraction peak shifted toward small angles, indicating Mo^6+^ stochastically located at the interstitial sites of the matrix lattice as a solution element.

**Figure 2:**
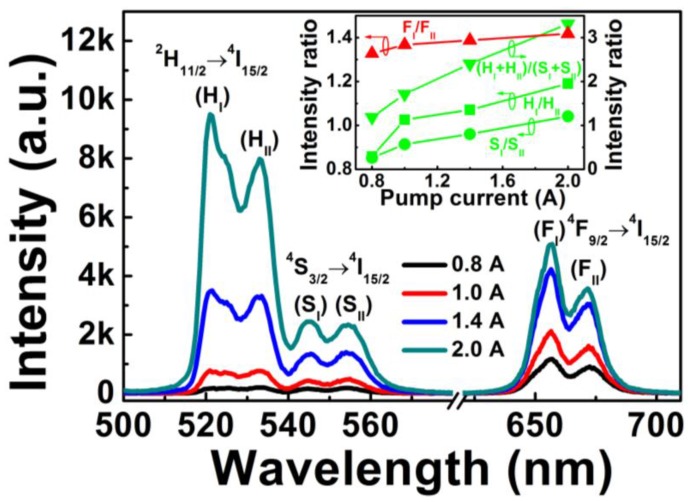
Upconversion emissions spectra of Er^3+^-Yb^3+^-Mo^6+^-codoped TiO_2_ with different pump currents. Inset shows corresponding upconversion emission intensity ratios *versus* the pump current.

Figure 2 shows the upconversion emission spectra of Er^3+^-Yb^3+^-Mo^6+^-codoped TiO_2_ under different pump currents. Green and red upconversion emissions were observed in the wavelengths of 500–540 nm, 540–580 nm, and 620–710 nm, corresponding to ^2^H_11/2_→ ^4^I_15/2_, ^4^S_3/2_→ ^4^I_15/2_, and ^4^F_9/2_→ ^4^I_15/2_ transitions of Er^3+^ ions, respectively. Each transition (^2^H_11/2_→ ^4^I_15/2_, ^4^S_3/2_→ ^4^I_15/2_, and ^4^F_9/2_→ ^4^I_15/2_) was divided into two emission peaks, which indicated ^2^H_11/2_, ^4^S_3/2_, and ^4^F_9/2_ levels of Er^3+^ split into three coupled Stark sublevels of ^2^H_11/2(I)_·(H_I_) and ^2^H_11/2(II)_·(H_II_), ^4^S_3/2(I)_·(S_I_) and ^4^S_3/2(II)_·(S_II_), and ^4^F_9/2(I)_·(F_I_) and ^4^F_9/2(II)_·(F_II_), respectively, due to the effect of crystal field environment on Er^3+^ ions. As the LD pump current increased from 0.8 to 2.0 A, the position and number of upconversion emission peaks did not change, whereas the intensity of green and red emissions markedly increased due to the increase in excitation power.

The inset in Figure 2 shows the upconversion emission intensity ratios of H_I_/H_II_, S_I_/S_II_, F_I_/F_II_, and (H_I_ + H_II_)/(S_I_ + S_II_) *versus* the pump current. All intensity ratios of H_I_/H_II_, S_I_/S_II_, F_I_/F_II_ and (H_I_ + H_II_)/(S_I_ + S_II_) increased alongside the pump current, implying that the nonradiative processes of Er^3+^ in Er^3+^-Yb^3+^-Mo^6+^-codoped TiO_2_ phosphor can partially transform pump energy into heat energy, therefore elevating the phosphor temperature. The temperature variation induced by increasing the pump current caused changes in the intensity ratio [19]; this suggests that the temperature-dependent intensity ratio for the four coupled energy levels of H_I_/H_II_, S_I_/S_II_, F_I_/F_II_, and (H_I_ + H_II_)/(S_I_ + S_II_) can be utilized for optical temperature sensing.

**Figure 3:**
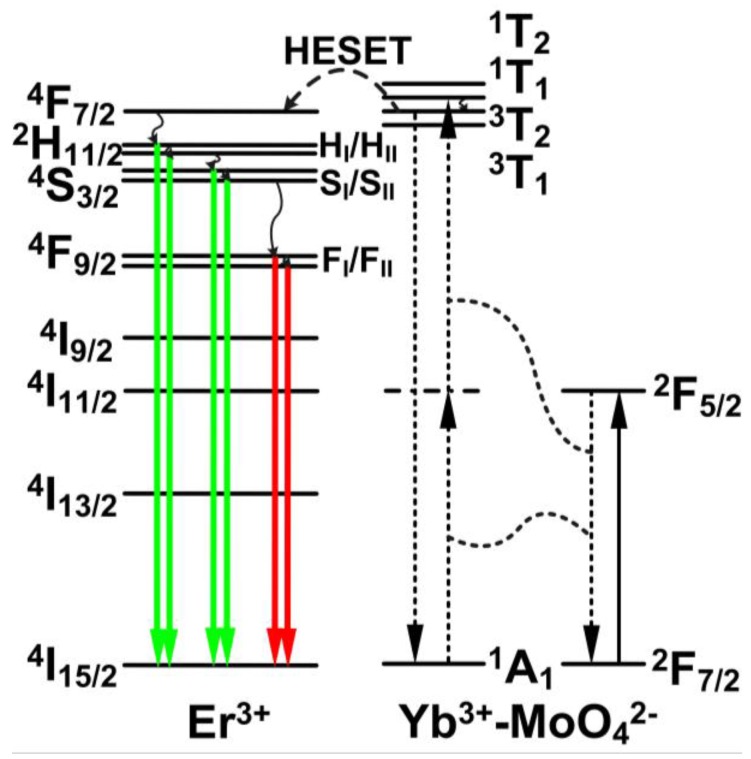
Schematic energy level diagram of Er^3+^-Yb^3+^-Mo^6+^-codoped TiO_2_ phosphors under 976 nm LD excitation. Wavy arrows indicate nonradiative relaxation.

Figure 3 shows a schematic energy level diagram of the Er^3+^-Yb^3+^-Mo^6+^-codoped TiO_2_ phosphors under 976 nm LD excitation. The upconversion mechanism of Er^3+^ after the addition of Mo^6+^ was reported in a previous study on the sensitization of the Yb^3+^-MoO_4_^2−^ dimer to Er^3+^ [20,21,22]. Through a cooperative sensitization process in the Yb^3+^-MoO_4_^2−^ dimer, two excited Yb^3+^ ions nonradiatively transfer their energy to MoO_4_^2−^. This process is followed by a high excited state energy transfer (HESET) to the ^4^F_7/2_ level of Er^3+^ ions. After nonradiative relaxations from ^4^F_7/2_ to the Stark sublevels of H_I_, H_II_, S_I_ and S_II_, green upconversion emissions are produced by transitions of H_I_/H_II_/S_I_/S_II_→ ^4^I_15/2_. The nonradiative relaxation from S_II_ to F_I_ and F_II_ levels and subsequent transitions of F_I_/F_II_→ ^4^I_15/2_ generate red emissions.

**Figure 4:**
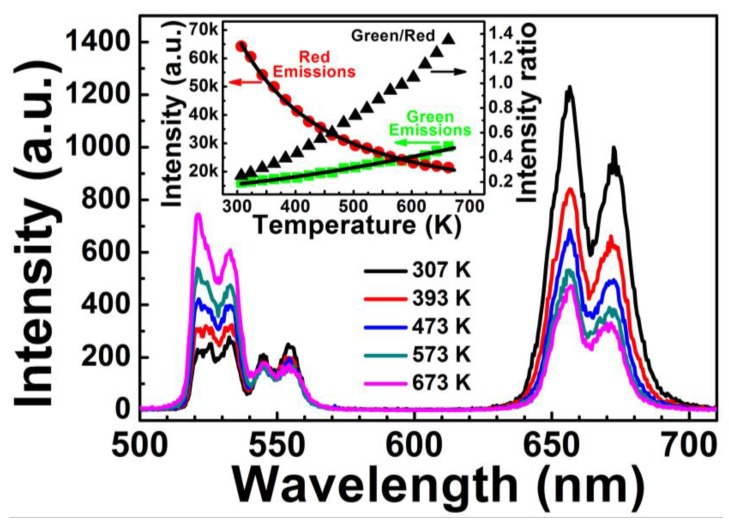
Upconversion emissions spectra of Er^3+^-Yb^3+^-Mo^6+^-codoped TiO_2_ at different temperatures. Inset shows the integrated intensity of green and red emissions and the intensity ratio of green to red emissions as a function of temperature. The solid lines for the temperature-dependent intensity of red and green emissions are fitting curves by Equations (1) and (2).

In order to distinguish the effects of temperature from the pump current on the intensity ratio (Figure 2), the upconversion emission properties of Er^3+^-Yb^3+^-Mo^6+^-codoped TiO_2_ were measured under different temperatures. Figure 4 shows the upconversion emissions spectra of Er^3+^-Yb^3+^-Mo^6+^-codoped TiO_2_ at measured temperatures between 307 and 673 K. Changes in temperature had no influence on the bands of green and red emissions from ^2^H_11/2_/^4^S_3/2_→ ^4^I_15/2_ and ^4^F_9/2_→ ^4^I_15/2_ transitions of Er^3+^ between 500 to 580 nm and 620 to 700 nm, respectively; the intensity varied with temperature, however. The inset in Figure 4 shows the intensity of green and red emissions and the intensity ratio of green to red emissions as a function of temperature. The intensity of red emissions decreased with increasing temperature, in accordance with the classical theory of thermal quenching. Temperature-dependent intensity of the red emissions can be expressed as follows [23]:

(1) $$I\left(T\right)=\frac{I\left(0\right)}{1+A\exp\left(-\Delta E^{\prime }/kT\right)}$$

where *T* is the absolute temperature, and *I*(*T*) and *I*(0) are the fluorescence intensities at temperatures of *T* and 0 K, respectively; Δ*E*′ is the activation energy, *k* is the Boltzmann constant, and *A* is a constant. The temperature-dependent intensity of red emissions fits well to Equation (1), where Δ*E*′_(FI+FII)_ = 0.074 eV.

Conversely, the intensity of green emissions increased with increasing temperature, which does not satisfy the classical theory of thermal quenching, likely due to the increased Yb^3+^ absorption cross-section at elevated temperatures [22,24]. A general theoretical description of the green upconversion emission can be given by [22]:

(2) $$I_{green}=B{\left[1-\exp\left(-\frac{h\nu }{kT}\right)\right]}^{-2}$$

where *B* is a constant, and *hν* is the phonon energy participating in the multiphonon-assisted excitation. The dependence of green upconversion emissions on temperature fits well to Equation (2). The *I_green_*/*I_red_* value increased with temperature, causing the color to turn from red to green with elevated temperature.

**Figure 5:**
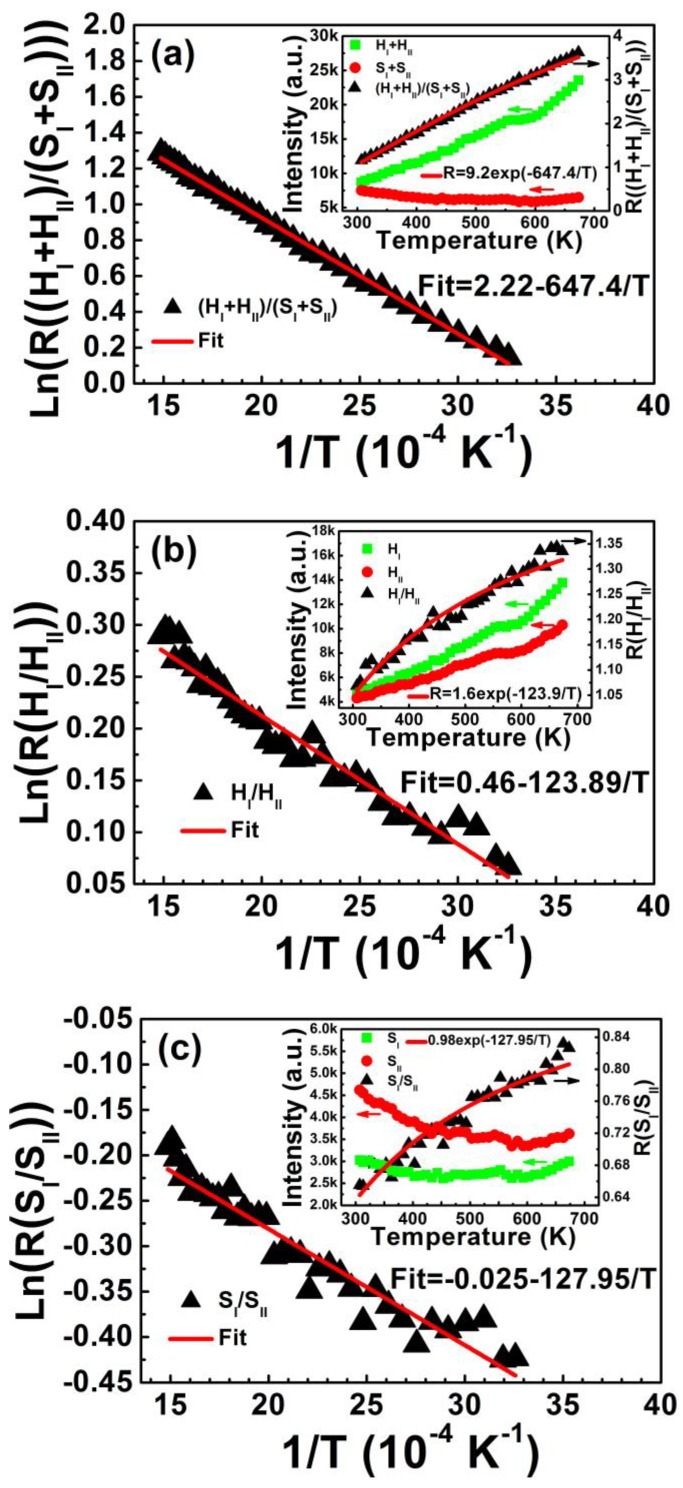

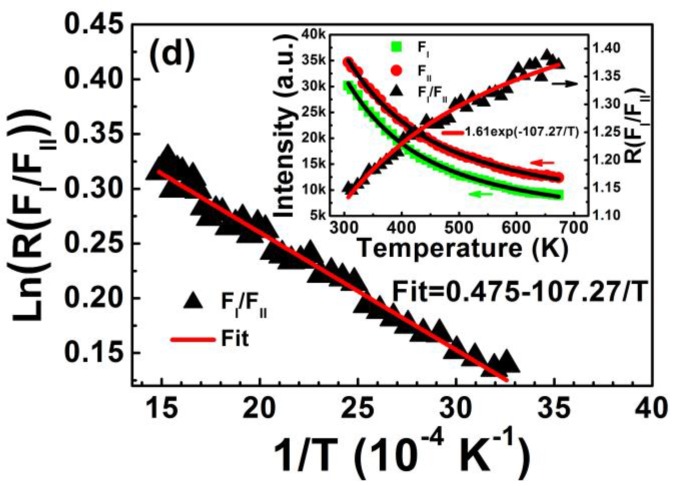
FIR plots of (**a**) (H_I_ + H_II_)/(S_I_ + S_II_); (**b**) H_I_/H_II_; (**c**) S_I_/S_II_; and (**d**) F_I_/F_II_ as a function of inverse temperature in the range of 307–673 K. Insets show corresponding upconversion emission intensity and intensity ratio relative to temperature. FIR plots are fitted by Equation (3) and the temperature-dependent intensities of red emissions in (**d**) are fitted by Equation (1).

According to previous research [1], the relative population of two “thermally coupled” energy levels with separation of the order of thermal energy follows a Boltzmann-type population distribution, causing variation in the transitions of two closely spaced levels at elevated temperature if pumped through a continuous light source. After populations are thermalized at two closely spaced levels, the FIR of upconversion emissions (*R*) related to the transitions of both levels can be written as follows:

(3) $$R=\frac{I_{\text{upper}}}{I_{\text{lower}}}=\frac{N_{\text{upper}}}{N_{\text{lower}}}=C\exp\left(\frac{-\Delta E}{kT}\right)$$

where *I*_upper_, *I*_lower_, *N*_upper_, and *N*_lower_ are the fluorescence intensity and number of ions for the upper and lower thermalizing energy levels, respectively; Δ*E* is the energy gap between two coupled levels, and *C* is a constant relative to the degeneracy, emission cross-section, and angular frequency of corresponding transitions. Equation (3) suggests that FIR is related to the energy gap Δ*E* and temperature *T*. Figure 5 shows FIR plots of (H_I_ + H_II_)/(S_I_ + S_II_), H_I_/H_II_, S_I_/S_II_, and F_I_/F_II_ as a function of inverse absolute temperature from 307 to 673 K. The inset shows corresponding upconversion emission intensity and the intensity ratio relative to temperature. The experimental data fits well to Equation (3). Energy gaps Δ*E* of the four coupled energy levels of (H_I_ + H_II_)/(S_I_ + S_II_), H_I_/H_II_, S_I_/S_II_, and F_I_/F_II_ are calculated in Table 1. The decreased intensity of two red emissions with elevated temperature, shown in the inset of Figure 5d, can also be fitted to Equation (1). The activation energy of F_I_ and F_II_ levels is calculated as Δ*E*′_FI_ = 0.069 eV and Δ*E*′_FII_ = 0.080 eV, which is consistent with the average activation energy of (F_I_ + F_II_) level (Δ*E*′_(FI+FII)_ = 0.074 eV) shown in Figure 4.

**Table 1:** Energy gap of coupled energy levels Δ*E*, pre-exponential factor *C*, maximum sensitivity *S*_max_, temperature of maximum sensitivity *T*_max_ and upconversion emission intensity for the four coupled energy levels of (H_I_ + H_II_)/(S_I_ + S_II_), H_I_/H_II_, S_I_/S_II_ and F_I_/F_II_.

Coupled Energy Levels	(H_I_ + H_II_)/(S_I_ + S_II_)	H_I_/H_II_	S_I_/S_II_	F_I_/F_II_
Δ*E* (eV)	0.0558	0.0107	0.0110	0.0093
*C*	9.2	1.6	0.98	1.61
*S*_max_ (10^−^^4^·K^−^^1^)	76.7	69.7	41.4	81.0
*T*_max_ (K)	324	62	64	54
Upconversion intensity	Higher	Higher	Low	Highest

For optical temperature-sensing applications, it is crucial to know the rate at which the FIR changes with temperature, known as the absolute sensitivity *S_a_*, which is expressed as follows [1]:

(4) $$S_{a}=\frac{1}{R}\frac{dR}{dT}=\frac{\Delta E}{kT^{2}}$$

Equation (4) makes clear that the appropriate selection of two thermally coupled energy levels with a suitable energy difference Δ*E* is very important. Larger Δ*E* benefits absolute sensitivity and accurate measurement of emission intensity, due to the decrease of fluorescence peak overlap originating from the two individual thermally coupled energy levels. Knowing this, the absolute sensitivity *S*_a_ when using coupled energy levels of (H_I_ + H_II_)/(S_I_ + S_II_) (with the largest possible Δ*E* = 0.0558 eV) is higher than those using the other three coupled levels, as shown in Table 1. The energy gap Δ*E* must be not too large, though, or thermalization no longer occurs.

Considering practical applications, it is extremely useful to be aware of variations in sensitivity with temperature. Relative sensitivity *S_r_* is expressed [25]:

(5) $$S_{r}=\frac{dR}{dT}=R\frac{\Delta E}{kT^{2}}$$

**Figure 6:**
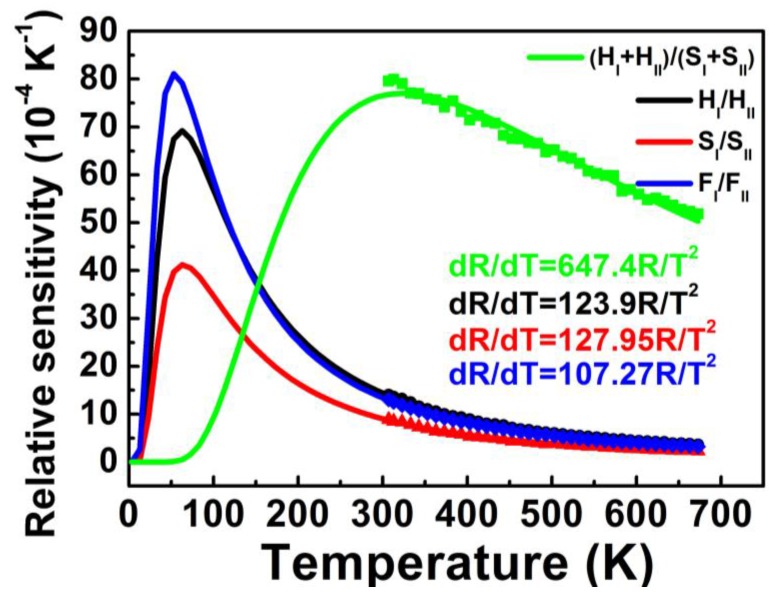
Relative sensitivities *S_r_* as a function of temperature for the four coupled energy levels of (H_I_ + H_II_)/(S_I_ + S_II_), H_I_/H_II_, S_I_/S_II_ and F_I_/F_II_. Closed symbols are the experimental data and the lines are the theoretical values calculated by Equation (5).

Compared to absolute sensitivity *S_a_*, relative sensitivity *S_r_* is dependent on not only energy gap Δ*E*, but also the intensity ratio FIR. Equation (3) indicates that larger FIR causes larger *C*. Thus, larger Δ*E* and FIR (or *C*) contribute to higher *S_r_*. Table 1 also shows pre-exponential factor *C* values for the four pair energy levels (H_I_ + H_II_)/(S_I_ + S_II_), H_I_/H_II_, S_I_/S_II_, and F_I_/F_II_. The coupled energy levels of (H_I_ + H_II_)/(S_I_ + S_II_) processed larger relative sensitivity *S*_r_ than those of H_I_/H_II_, F_I_/F_II_, or S_I_/S_II_. *S_r_* as a function of temperature for the four coupled energy levels calculated by Equation (5) is shown in Figure 6, in accordance with the above results in the measured temperature range 307–673 K.

Maximum sensitivity *S*_max_ and temperature *T*_max_, at which the sensor has maximum sensitivity *S*_max_, are of utmost importance because these two parameters indicate the highest sensitivity properties and optimum operating temperature range of optical thermal sensors. According to Equation (5), *S*_max_ and *T*_max_ can be calculated by $dS_{r}/dT=0$ as follows:

(6) $$S_{\max}=\frac{0.54C}{\Delta E/k}$$

(7) $$T_{\max}=\frac{1}{2}\frac{\Delta E}{k}$$

Equation (6) indicates that a larger pre-exponential factor *C* and smaller energy difference Δ*E* of coupled energy levels help to increase *S*_max_. Equation (7) shows that *T*_max_ is relative to the energy difference Δ*E*, in which the sensor with a larger Δ*E* has a higher *T*_max_. *S*_max_ and *T*_max_ for the four coupled energy levels are shown in Table 1. The highest *T*_max_ was found for (H_I_ + H_II_)/(S_I_ + S_II_) coupled energy levels used for thermal sensing, due to a larger Δ*E*. The relatively larger *C* and smallest Δ*E* in F_I_/F_II_ coupled energy levels used for thermal sensing resulted in the highest sensitivity *S*_max_.

Temperature measurement error can be calculated using the relation [8,26]:

(8) $$\Delta T=\Delta R\frac{kT^{2}}{R\Delta E}=\frac{\Delta R}{S_{r}}$$

Larger *S*_r_ and smaller Δ*R* imply better accuracy. As shown in Figure 6, larger *S_r_* at a higher temperature for coupled energy levels of (H_I_ + H_II_)/(S_I_ + S_II_) led to a better accuracy in the high temperature range. Likewise, better accuracy can be expected in the low temperature range using H_I_/H_II_, S_I_/S_II_ and F_I_/F_II_ coupled energy levels for thermal sensing.

The separation of two coupled energy levels Δ*E* should be large enough to avoid overlap of the two fluorescence emissions and to produce efficient luminescence for feasible and accurate intensity measurement. The efficient luminescence of Er^3+^-doped materials also contributes to the ready detection of luminescence and Δ*R* accuracy, where only low excitation power is needed. Table 1 shows where (H_I_ + H_II_)/(S_I_ + S_II_) coupled energy levels had the highest accuracy of all samples, due to a larger Δ*E* and the strongest luminescence intensity; conversely, S_I_/S_II_ coupled energy levels had the lowest accuracy, evidenced by a smaller Δ*E* and the lowest luminescence intensity, which are altogether consistent with the results shown in Figure 5.

## 4. Conclusions

The green and red upconversion emissions by transitions of Er^3+^ Stark sublevels were observed in Er^3+^-Yb^3+^-Mo^6+^-codoped TiO_2_ phosphors in this study. There are four coupled energy levels of Er^3+^ ions due to the effect of the crystal field environment on Er^3+^, each of which was utilized to study temperature-dependent upconversion emission properties. Based on the FIR technique, the optical temperature-sensing behaviors of sensing sensitivity, measurement error, and operating temperature for the four coupled energy levels were discussed in detail, with all closely related to the energy gap of the coupled energy levels, FIR value, and luminescence intensity. High sensitivity and negligible error are obtainable through the use of different coupled energy levels for optical sensing, throughout a wide range of temperature in an independent system. The utilization of coupled energy levels by Stark split is a new and effective method in the realization of multiple optical temperature measurement.
